# An Emergency Adaptation of Anterolateral and Clamshell Thoracotomy for Blunt Traumatic Right Atrial Rupture: A Case Report

**DOI:** 10.7759/cureus.49208

**Published:** 2023-11-21

**Authors:** Doaa Alfraidy, Fayez G Aldarsouni, Hatoon Dagestani, Ghassan Z Al Ramahi

**Affiliations:** 1 Department of Surgery, King Saud University Medical City, Riyadh, SAU; 2 Department of Trauma Surgery, King Saud Medical City, Riyadh, SAU

**Keywords:** sternotomy, mediastinal trauma, anterolateral thoracotomy, clamshell thoracotomy, cardio thoracic surgery, emergency critical care, acute care surgery and trauma, intraop bleeding, blunt cardiac trauma, bilateral anterolateral thoracotomy

## Abstract

The high mortality rate of blunt cardiac injuries is primarily due to the condition's severity and the challenges associated with pre-hospital survival. The absence of definitive diagnostic modalities necessitates prompt and adaptable surgical intervention. We present an 18-year-old male who sustained a right atrial blunt traumatic cardiac rupture following a motor vehicle collision. Despite initial stabilization with blood products and vasopressors and the necessitated emergent surgical exploration, the case required various surgical techniques, including anterolateral followed by an extension to a clamshell thoracotomy and laparotomy to manage the complex cardiac rupture and associated injuries. Furthermore, it underscores the critical nature of surgical incision in such patients and its impact on the overall prognosis. The successful outcome, highlighted by intraoperative decision-making and proper postoperative care, demonstrates that with timely and adaptable surgical approaches, even the most severe cases of traumatic blunt cardiac ruptures can be managed effectively.

## Introduction

Blunt cardiac injury (BCI) is a rare clinical entity with an incidence ranging from 0.16% to 2%, primarily due to the high mortality rate associated with the condition before patients can reach the emergency department (ED) [1]. The incidence of cardiac trauma reported varies significantly, from 8% in autopsy studies to 76% in clinical series [2,3]. This condition is not only life-threatening but also presents increased risks when accompanied by other traumatic injuries. Concurrent injuries to great vessels and abdominal organs are common, reported in up to 40% and 20% of cases, respectively [2]. The absence of gold-standard diagnostic modalities necessitates timely interventions [3]. When the patient is hemodynamically stable, electrocardiography (ECG) and computed tomography (CT) scans may be employed [4]. For patients presenting with signs of instability, adherence to Advanced Trauma Life Support (ATLS) protocols, clinical suspicion, echocardiography, and surgical exploration under general anesthesia via sternotomy or thoracotomy is crucial [2]. The pathophysiological mechanisms leading to blunt cardiac rupture remain poorly understood [1]. It is hypothesized that the injury results from a rapid increase in intracardiac pressure due to direct trauma increased venous pressure from abdominal and lower extremity compression, or the heart being compressed between the sternum and spine during deceleration, most commonly in motor vehicle collisions (MVC) [1,3,5]. In light of these challenges, we report a case of an 18-year-old male who sustained blunt cardiac trauma resulting in right atrial cardiac rupture. This report discusses the approach to diagnosis and intraoperative management for such patients, providing a detailed account of the clinical presentation, management strategies, and outcomes.

## Case presentation

**Figure 1:**
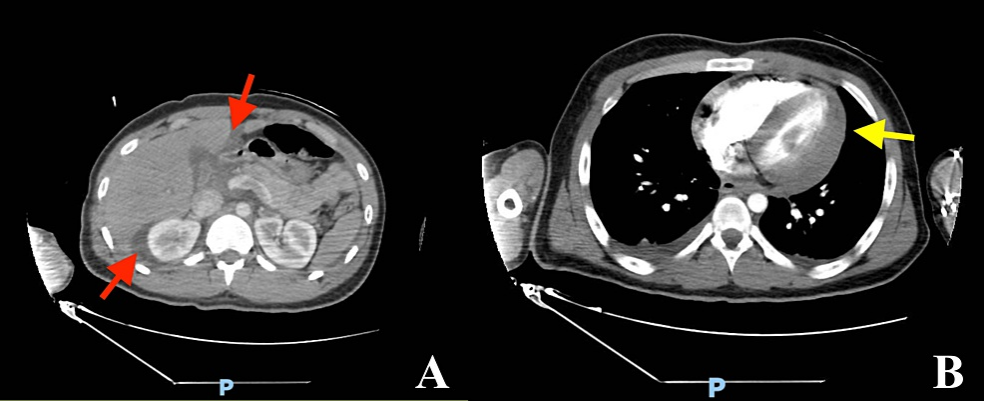
Axial Sections of Computed Tomography Scan of the Abdomen (A) and Chest (B) (A) Free fluid in the abdomen (red arrows). (B) Pericardial effusion (yellow arrow).

We report a case of an 18-year-old male with no significant medical history who was transferred to the ED of King Saud Medical City (KSMC), a major level 1 trauma center in the region. The patient was referred from a rural hospital approximately 180 minutes away from KSMC, following an MVC with high-impact and rapid deceleration. Upon initial presentation, the patient was intubated in the ED due to a deteriorating Glasgow Coma Scale and unstable blood pressure of 100/60 mmHg on two vasopressors, norepinephrine and dopamine. This prompted the activation of a Trauma Code, and our team managed the case. An Extended Focused Assessment with Sonography in Trauma (E-FAST) revealed positive findings for pericardial effusion and hemoperitoneum. Venous blood gas analysis indicated ongoing bleeding (pH: 7.23, lactate: 4 mmol/L). The patient was resuscitated with two units of packed red blood cells (pRBC) and four units of fresh frozen plasma (FFP), allowing for the cessation of vasopressors. After achieving adequate hemodynamic stability, an ECG was performed, revealing a prolonged QT interval. A contrast-enhanced CT scan showed intrabdominal fluid (Figure 1A), associated with a grade 3 liver injury, fluid around the pericardium (Figure 1B), necessitating surgical exploration as a category 1 emergency surgery.

**Figure 2:**
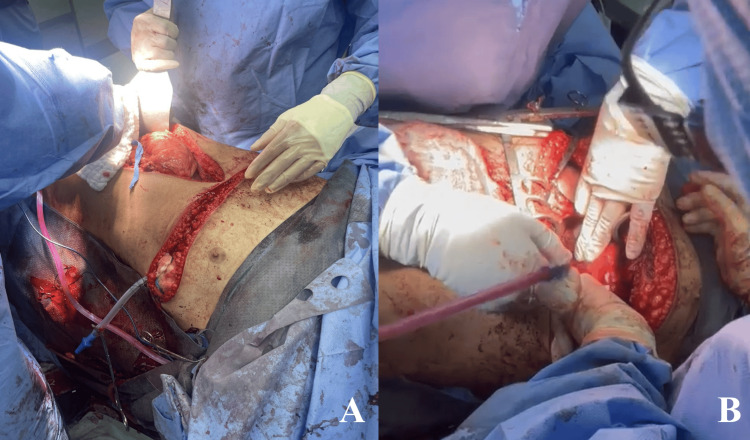
Intraoperative Distal View of the Clamshell Thoracotomy and Concomitant Laparotomy Incision (A) Extension of the thoracotomy incision visualized post-first layer closure. (B) Prior to closure, thorough irrigation is performed by the assistant during the cardiac injury closure.

In the operating room (OR), a 15 cm midline incision was made to explore the abdomen (Figure 2A). Examination of the bowel loops, liver, spleen, and other abdominal organs was done. A small 2 cm liver laceration at segment 4B was discovered and controlled with perihepatic packing, followed by closure using Surgicel® (Ethicon, Inc., Johnson & Johnson, NJ, USA) (a topical hemostatic agent). To address the suspected cardiac tamponade, a horizontal 5 cm incision below the xiphoid process was made, and a subxiphoid pericardial window was performed, releasing approximately 100 mL of blood. The patient's hemodynamics initially improved after relieving the tamponade. However, instability resumed as blood continued to flow, prompting a left anterolateral thoracotomy due to further deterioration of vital parameters. A 1.5-2 cm hole in the right atrial posterior wall was visualized and temporarily controlled with the index finger during thoracotomy. The patient experienced cardiac arrest, and a Satinsky vascular clamp was applied. Cardiac massage and transfusion of 14 units of pRBC were performed, with the return of spontaneous circulation achieved after 10 minutes of cardiac massage and defibrillation shocks. Due to arrhythmia and the challenging injury location, accessing the atrial hole for closure was difficult.

**Figure 3:**
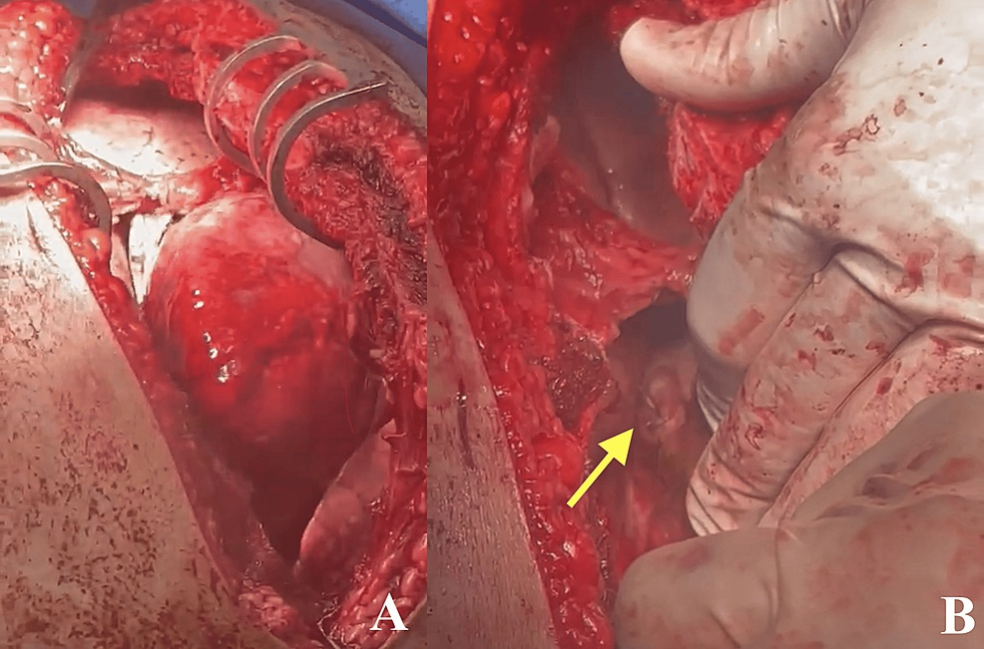
Intraoperative Proximal View of the Thoracotomy Incision (A) A closer look at the clamshell thoracotomy shows using Badgley Rib Spreader. (B) The closed cardiac injury on the posterior side of the right atrium, sutured with Prolene 3-0 (yellow arrow).

The thoracotomy was extended to a clamshell incision (Figures 2A, 2B, 3A), allowing closure of the injury with 3-0 Prolene (Ethicon, Inc., Johnson & Johnson, NJ, USA) and a pericardium pledget (Figure 3B). Internal mammary artery proximal and distal ends were ligated. Intercostal drains were inserted on both sides of the incision (Figure 2A), and the left pleural cavity was packed due to oozing from the intercostal muscles, necessitating a second look.

**Figure 4:**
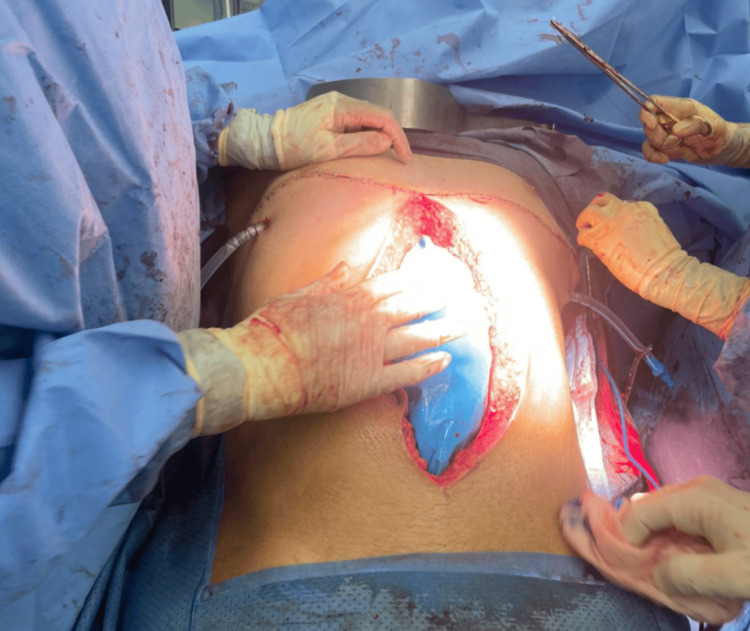
Intraoperative Distal View of the Closed Clamshell Incision and Temporary Closure of the Laparotomy Incision

The abdomen was temporarily closed (Figure 4), and the patient was admitted to the intensive care unit (ICU) for hemodynamic stabilization. During this time, the patient received two units of platelets and four units of FFP. Seventy-two hours later, the patient underwent definitive abdominal closure. Postoperatively, the patient remained in the ICU for 30 days with no complications except for electrolyte imbalances and ileus, which were managed conservatively. On postoperative day 30, the patient was downgraded to a ward, having regained consciousness and memory. Subsequently, on day 40, the patient was discharged in good health.

## Discussion

Directing the focus to the heart can be a pivotal process in the presentation of trauma. We began managing this patient by concentrating on ATLS principles due to his instability. As part of the E-FAST, we detected fluid around the heart. At that time, our attention was broader than the heart alone, as our examination concluded an associated hemoperitoneum. Thus, after stabilizing the patient, we proceeded with a CT scan for better visualization. With our focus on relieving the ongoing tamponade and examining the suspected associated injuries, we took the patient to the OR.

In the literature, we found the proposed algorithm by Jose E et al., published in 2012, and Eiferman's report in "Principles and Practice of Cardiothoracic Surgery" highly conclusive for the diagnosis of such patients [1,2]. Given the absence of gold-standard diagnostic modalities for BCI and the high mortality rate these patients carry, our highest priority in unstable traumatic patients was focusing on ATLS protocols. The interrelation between ATLS protocols and the Eastern Association for the Surgery of Trauma (EAST) guidelines for the management of BCI in the context of initial care is evident [2].

We decided to take the patient to the OR for a laparotomy after a CT scan showed fluid around the pericardium and confirmed the suspected lacerations in the liver and spleen. With the intention to relieve the tamponade, the rupture was not clear on the CT scan at that time. That's why we chose to examine the abdomen before the chest, as the patient's hemodynamics were maintained, and we suspected that the abdominal injury was the primary concern. After managing the liver laceration, we opened a pericardial window to relieve the tamponade. However, the bleeding persisted, and the patient's hemodynamics began to deteriorate. In our case, cardiac arrest occurred after we opened the chest for a pericardial window, necessitating a resuscitative thoracotomy [6].

**Figure 5:**
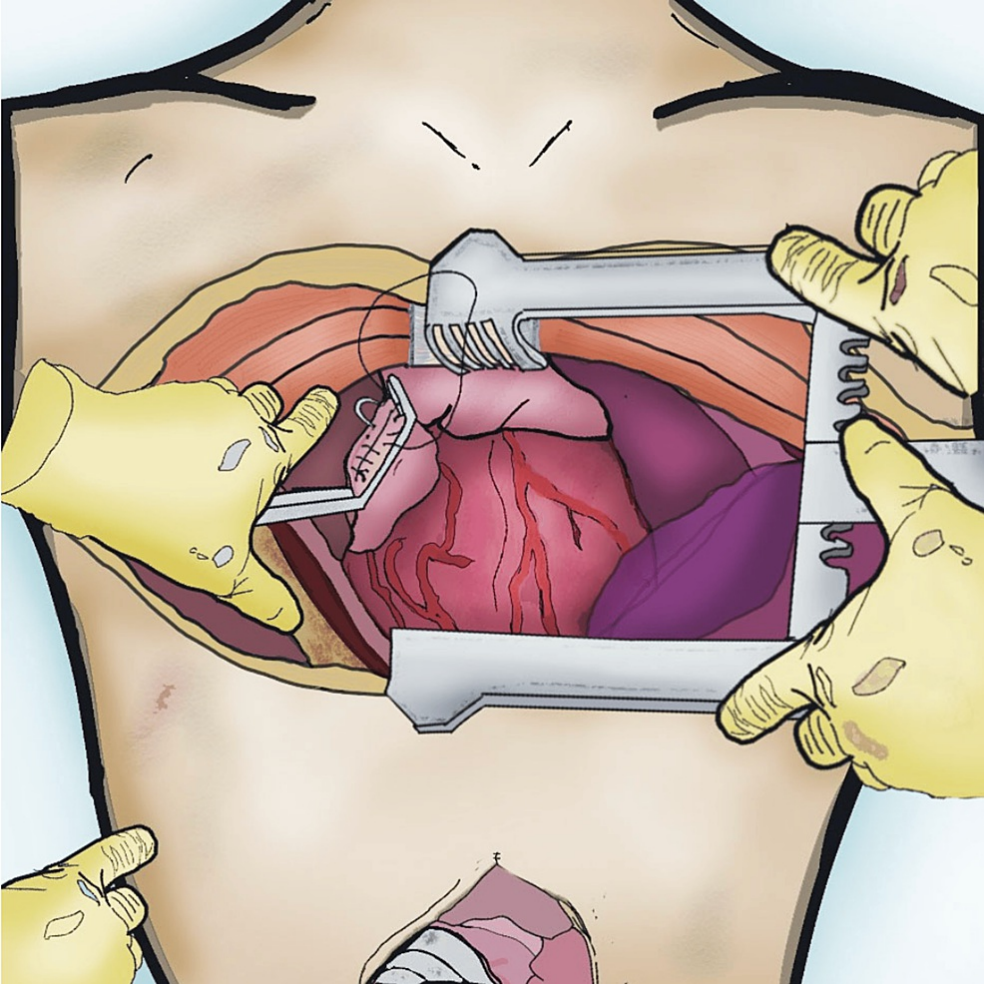
Illustration of the Repair Process for the Atrial Cardiac Rupture The injury was located in the atrial appendage, a vulnerable area on the posterior wall of the right atrium. Closure should typically performed after controlling atrial bleeding with a vascular clamp. In this patient, a Satinsky vascular clamp was utilized. Illustration by Fayez G. Aldarsouni

The hemodynamic collapse followed by cardiac arrest was a stressful and unexpected encounter. In such critical moments, as mentioned in "Top Knife," the adage "Putting brain in gear before knife in motion" becomes a determinant of the patient's path and prognosis. This is often more feasible for experienced surgeons; in such moments, panic could cloud thinking. Maintaining a systematic, algorithmic approach is essential in managing traumatic cardiac arrest. We performed an anterolateral thoracotomy that was further extended to a clamshell thoracotomy (Figure 5). This was done for better access to the injury. We used a Badgley Rib Spreader instead of the typically used Finochietto retractor, as it was not available at the moment (Figure 1A). There is still debate over the type of incision used [6,7]. Although median sternotomy has advantages over clamshell incisions in targeting specific injuries and minimizing morbidities, especially in the superior mediastinum [7], a sternotomy set is not always available in emergency settings. Thus, this advantage could only apply to elective settings. Moreover, in arresting situations when there is uncertainty about the exact location of the injury and potential associated great vessel injuries, and with the lack of luxury of time - as in our patient - a clamshell incision carries superiority in emergency settings [6]. Predicting the need to extend the incision prior to incising, as hemodynamics were gradually deteriorating, we performed an anterolateral thoracotomy instead of a sternotomy to allow for such a possibility. We ligated the internal mammary artery - also called the internal thoracic artery - and we advise this practice before closing the chest.

This patient was a young, previously healthy individual, and we believe this aided his postoperative course. Typically, a clamshell incision can carry risks related to pain and respiratory complications, increased length of stay, and increased operative time [7,8]. The healthy profile of our patient contributed to our decision-making at the moment; however, the increased postoperative length of stay was unanticipated for such a surgery.

## Conclusions

This case highlights the critical role of surgical adaptability in the management of suspected traumatic BCI. Prompt decision-making, coupled with the versatility of surgical technique, can significantly influence outcomes in traumatic cardiac emergencies.
